# Electrophysiologically distinct bed nucleus of the stria terminalis projections to the ventral tegmental area in mice

**DOI:** 10.3389/fncir.2022.1081099

**Published:** 2023-01-09

**Authors:** Yuka Miura, Mary Regis Shanley, Arabella Soriano Sanchez, Allyson K. Friedman

**Affiliations:** ^1^Department of Biological Sciences, Hunter College of the City University of New York, New York, NY, United States; ^2^Program in Biology, Graduate Center of the City University of New York, New York, NY, United States

**Keywords:** BNST, ventral tegmental area, GABA, *in vitro*, whole-cell patch-clamp recording, electrophysiology

## Abstract

The bed nucleus of the stria terminalis (BNST) is a highly heterogeneous limbic forebrain structure that serves as a relay connecting autonomic, neuroendocrine and behavioral function. It can be divided into over 16 individual subregions with distinct neuronal subpopulations based on receptors, transmitters, and neuropeptides. Specifically, the BNST projection to the ventral tegmental area (VTA), the dopamine hub of the brain, has been shown to have a crucial role in the stress response. However, in mice there is a lack of unbiased data on the functional diversity of this sub-population which serves as an upstream input to the VTA. The dopaminergic neurons in the VTA modify their ion channel activity and intrinsic membrane properties to adapt to stress in part from inputs from BNST projections. Therefore, we aimed to perform a multi-component characterization of the functional diversity of the BNST-VTA pathway. We studied the passive and active electrophysiological properties of virally identified population of BNST neurons that project to the VTA. We used a comprehensive series of *in vitro* recordings of electrophysiological variables and performed hierarchical clustering to determine the functional diversity of the projection neurons in the BNST-VTA pathway. Our study revealed four subpopulations in the BNST-VTA pathway, all of which differ in their activation profiles and likely have distinct inputs and function in the VTA. Our results will help resolve the discord in interpretation of the various roles of this electrophysiologically diverse projection and builds a foundation for understanding how the different neuronal types integrate signals.

## Introduction

The bed nucleus of the stria terminalis (BNST) is a key structure in the limbic system, playing a well-established role in anxiety and stress-reward interaction. The projection from the BNST to the ventral tegmental area (VTA) is a critical neural pathway involved in reward processing (Jennings et al., 2013), and is extensively studied in regards to drug and alcohol addiction (Dumont et al., 2008; Briand et al., 2010; Sartor and Aston-Jones, 2012) and anxiety (Jennings et al., 2013; Kim et al., 2013). Specifically, GABA inputs from the BNST onto the VTA have been found to be necessary to promote binge-like ethanol intake (Companion and Thiele, 2018), which can be strengthened by stress (Ostroumov et al., 2016). It has also been shown that self-administration of cocaine, drug-seeking behavior (Aston-Jones and Harris, 2004; Dumont et al., 2005; Grueter et al., 2006; Jalabert et al., 2009), and stress-induced reinstatement of cocaine place preference (Briand et al., 2010) are regulated by the BNST-VTA circuit.

While the BNST is composed primarily of GABAergic neurons, it has heterogeneous neuronal types that are different in their intrinsic membrane properties. BNST neurons have been distinguished into Type I, Type II, and Type III, and Type “Others” based on discrimination of the firing pattern in response to depolarizing/hyperpolarizing current injection in rats (Hammack et al., 2007). Depolarizing current injection causes regular firing in Type I, burst firing in Type II, and non-regular firing in Type III. Hyperpolarizing current injection induces a voltage sag in Type I, rebound spiking exclusively in Type II, but neither in Type III neurons (Hammack et al., 2007; Yamauchi et al., 2018). Type “Others” is identified by firing with a single spike (Rodríguez-Sierra et al., 2013; Silberman et al., 2013; Daniel et al., 2017; Ch’ng et al., 2019).

Membrane properties of these neurons set their response to stimuli, thereby affecting signal transduction to the next neuron in the circuit to determine an animal’s behavior. Altered membrane properties and changes in coherent communication between neurons are seen in rodent models of reward circuit-related disorders such as depression (Rosenkranz et al., 2010), drug abuse (Mu et al., 2010), and alcohol use disorder (Marty and Spigelman, 2012; Dao et al., 2021). Thus, understanding intrinsic membrane properties of neurons within BNST-VTA circuit is critical to identify precise targets for effective treatments for disorders in which interaction between reward and stress is profoundly involved.

Despite the importance of understanding the neuronal properties in this circuit, no study has identified if all subtypes project to the VTA in mice. Similarly, it is unknown if the cells maintain similar functional differences as described in rats. Research in rats found that 80% of VTA-projecting BNST neurons are Type III (Yamauchi et al., 2018) while one study in mice showed that there are no Type III neurons in the same projection (Silberman et al., 2013). Moreover, multiple studies have relied on visual discrimination to categorize the various neuronal types potentially biasing the classifications.

Given the discord in the literature, we sought to address two specific questions. First to determine what functional cell-types exist in the VTA-projecting BNST neurons in mice and second to establish an unbiased way to categorize the BNST neurons. In an effort to characterize VTA projecting BNST neurons, we used a retrograde viral vector with a green fluorescent protein to tag neurons in the BNST that project to VTA, and combined this with whole-cell patch clamp electrophysiological analysis. This allowed us to identify the electrophysiological properties specifically from the BNST neurons that project to the VTA.

Our findings identify four populations of neurons within the BNST-VTA projection that exhibit distinct electrophysiological properties: Type I, Type II, Type III, and Type “Others.” Utilizing an unbiased hierarchical clustering and heatmap visualization, we revealed shared properties and functional distinctions among these subpopulations.

## Materials and Methods

### Animals

All experiments were carried out in accordance with the National Institute of Health guidelines for the care and use of Laboratory animals and were approved by the Hunter College Institutional Animal Care and Use Committee (IACUC). Male C57Bl/6J mice (Jackson Laboratory) at age 9–10 weeks were used. All mice were housed four per cage and food and water were available *ad libitum*. Light/dark cycle was 12 h (8:30 AM/8:30 PM).

### Surgical preparation

Male C57Bl/6J mice (*N* = 24) at 9–10 weeks were anesthetized by intraperitoneal injection of a mixture of ketamine (100 mg/kg body weight) and xylazine (10 mg/kg body weight) and placed in a stereotaxic apparatus. After confirming that the mouse was deeply anesthetized by sensory stimulation, aseptic preparation was performed and a small incision was made on head skin. For retrograde labeling of BNST neurons projecting to the VTA, a 33-gauge needle of a 10 μl Hamilton syringe was filled with 0.3 μl of rAAV2 retro-hSyn-eYFP (5.1 × 10^12^ vg/ml; lot #:AV8221; UNC Vector Core Facility, Chapel Hill, NC; Tervo et al., 2016). The virus was bilaterally injected into the VTA (Angle 7°; A-P −3.2; L-M +1.0; D-V −4.6 (mm) from Bregma) at the injection speed of 0.1 μl/min. We waited 5 min to prevent backflow before removing the needles from the brain. After surgical procedures were performed, 500 μl of 0.09% NaCl saline and 0.0015 mg of Buprenorphine were injected subcutaneously for post-operative care. Mice were single-housed in a clean cage after the surgery.

### BNST coronal slice preparation

Slice preparation was performed as previously described (Friedman et al., 2016). Artificial cerebrospinal fluid (aCSF) was prepared in the following ion concentration; in (mM), NaCl 128; D-Glucose 10; NaH_2_PO_4_ 1.25; NaHCO_3_ 25; MgCl_2_ 2; KCl 3; CaCl_2_ 2. aCSF was ice-cold and oxygenated with 95% oxygen and 5% carbon dioxide. Two weeks after the viral injection surgery, 11–12 week-old mice were anesthetized with isoflurane (1-chloro-2,2,2-trifluoroethyl-difluoromethylether). After confirming that the mouse was deeply anesthetized with sensory stimulation, an incision was made on the chest. Ice-cold oxygenated aCSF was trascardially perfused prior to rapid decapitation. After harvesting the brain, the brain was blocked into BNST-containing and VTA-containing blocks. The BNST-containing block was fixed on the buffer tray of a Microslicer (Microslicer DTK-1000, Dosaka EM, Kyoto, Japan) and the VTA-containing block was soaked in 4% PFA in 1× PBS for *post-hoc* histology experiments. Acute brain slices containing BNST neurons were cut at 250 μm-thick in cold oxygenated sucrose aCSF [in (mM), sucrose 227; D-Glucose 10; NaH_2_PO_4_ 1.25; NaHCO_3_ 24; MgCl_2_ 2; KCl 3; CaCl_2_ 2] using the microslicer. The BNST slices were transferred into a recovery chamber with oxygenated aCSF for 1 h at 36°C. The recovery chamber is then moved to room temperature with continuous oxygenation and slices are used for recording for up to a 4 h period.

### Whole-cell patch-clamp recording

Recordings were performed at 37°C using an inline solution heater (SH-27B, Warner Instruments, Holliston, MA, USA) and temperature controller (TC-324C, Warner Instruments, Holliston, MA, USA). Slices were transferred to recording chamber that was continually perfused with oxygenated aCSF at a flow rate of 3.1 ml/min. Recording pipets were made from thick-walled borosilicate glass (BF150-86-10, Sutter Instrument, Novato, CA, USA). Glass pipets were pulled by P-97 Flaming/Brown micropipette puller (Sutter Instrument, Novato, CA, USA). Patch pipet for whole-cell voltage-clamp and current-clamp (3–8 mΩ) was filled with internal solution [in (mM), K-gluconate 115; KCl 20; MgCl_2_ 1.5; Phosphocreatine 10; K-ATP 2; Na-GTP 0.5; HEPES 10; pH 7.4, 284 mOsm]. BNST was identified by anatomical location guided by anterior commissure spanning both hemispheres and lateral ventricles. The BNST was visualized with 4× objective lens (PLN 4X, Olympus, Tokyo, Japan) and BNST neurons were visualized under infrared light with 40× objective (LUMPLFLN, Olympus, Tokyo, Japan) immersed in aCSF. eYFP-labeled neurons were visualized with a fluorescent lamp (X-Cite 120Q, Lumen Dynamics, Mississauga, ON, Canada) light with 470 nm filter and recordings were made from eYFP labeled neurons. Neurons of interest were identified with the presence of fluorescence in the soma. Neurons without fluorescence were not recorded. After the creation of giga-Ω seal, the cell membrane was ruptured by small suction to create whole-cell configuration. Resting membrane potential (RMP, mV) was recorded at *I* = 0 after whole-cell configuration was created. Excitability and maximum firing rate (MFR) were measured in current-clamp mode and *I*_h_ was measured in voltage-clamp mode using the Multiclamp 700 B amplifier (Molecular Devices, San Jose, CA, USA) and data acquisition was made in Axon Digidata 1550 B (Molecular Devices, San Jose, CA, USA).

### Recording protocol

#### Current-to-spike relationship (excitability)

In current clamp configuration, neurons were characterized by a series of hyperpolarizing and depolarizing current steps from a holding membrane potential of −60 mV. Voltage response to depolarizing/hyperpolarizing current was recorded from −80 pA to +60 pA for 500 ms in a 10 pA increment.

#### Hyperpolarization current (*I*_h_)

In voltage-clamp configuration, hyperpolarization activated current (*I*_h_) was measured with a series of hyperpolarizing injections from a holding membrane potential of −60 mV. *I*_h_ was recorded in −60 mV to −150 mV voltage steps for 800 ms in a 10 mV increment.

#### Maximum firing rate

In a current-clamp setting, maximum firing rate (MFR) was measured by recording the voltage response to a large depolarizing current from a holding membrane potential of −60 mV. Maximum firing rate (MFR) was evaluated from 50 pA to +750 pA for 1,000 ms in a 50 pA increment.

### Immunohistochemical methods

#### PFA perfusion

For BNST histology experiments, mice that were injected with rAAV2 retro-hSyn-eYFP were anesthetized with urethane intraperitoneal injection (1.5 g/kg body weight; Pagliardini et al., 2013). After confirming that the mouse was deeply anesthetized with sensory stimulation, a small incision was made on the chest. The right atrium was cut and 21 gauge needle filled with 1× PBS was inserted into the left ventricle to start transcardiac perfusion. After perfusion with 1× PBS, perfusion was switched to 4% PFA. Tremor by PFA fixation reaction was observed. The brain was extracted and stored in 4% PFA overnight.

#### Slicing of fixed brain

After storing the VTA-containing brain block or PFA-perfused brain in 4% PFA in 1× PBS overnight, it was transferred to 30% sucrose 1× PBS with 0.02% Na Azide. The brain was sliced at 30 μm on cold microtome (SM2010R, Leica) with dry ice. Slices were stored in 1× PBS with 0.02% Na Azide at 4°C.

#### Immunohistochemistry

For viral injection confirmation in the VTA, slices were first washed with 1× PBS three times for 10 min each. The slices were blocked with 1× PBS containing 0.3% Triton-X and 2% normal goat serum for 30 min, followed by free floating incubation on the shaker with primary antibody (AB9702, anti-TH raised in chicken, Millipore, 1:1,000, lot# 3519393) overnight at 4°C. The slices were washed with 1× PBS containing 0.3% Triton-X three times for each 10 min, followed by incubation on shaker with secondary antibody (A21449, Alexa Fluor 647 goat anti-chicken, Invitrogen, 1:600, lot# 2079903) for 1 h at room temperature. For visualization of retrogradely labeled neurons in the BNST, slices were first washed with 1× PBS once for 10 min. Slices were transferred to sodium citrate that was pre-heated at 80°C for 30 min. Once the solution was cooled to room temperature, slices were washed with 1× PBS. The slices were blocked with 1× PBS containing 0.3% Triton-X and 10% normal goat serum for 1 h, followed by incubation on shaker with primary antibody (A11122, anti-GFP raised in rabbit, Thermo Fisher, 1:500, lot# 2477546) in 1× PBS containing 0.3% Triton-X and 5% normal goat serum overnight at 4°C. Slices were washed with 1× PBS containing 0.3% Triton-X three times for each 15 min, followed by incubation on shaker with secondary antibody (A11008, anti-rabbit IgG raised in goat, Thermo Fisher, 1:400, lot# 2147635) in 1× PBS containing 0.3% Triton-X and 5% normal goat serum for 2 h at room temperature. The slices were washed with 1× PBS, 1× PBS containing 0.3 μM DAPI, and 1× PBS for 10 min each. The slices were mounted on microscope slides (Superfrost Plus, Fisherbrand, MA, USA) and dried overnight. Dehydration and clearing protocol were performed by immersing dried slices on slides in 70%, 90%, and 100% ethanol (200 proof ethanol, Decon Labs, PA, USA) for 2 min followed by soaking in xylene (Millipore, MA, USA) for 10 min twice. The slices were cover slipped (Rectangles, Fisherbrand) with Permount (Fisher Scientific, MA, USA).

### Confocal imaging

Images were acquired using a Nikon A1 laser-scanning confocal microscope (Nikon, Japan) with NIS-Elements AR software (version 4.60.00). eYFP was detected at an excitation wavelength of 488 nm, AlexaFluor 647 was detected at an excitation wavelength of 640 nm, and DAPI was detected at an excitation wavelength of 405 nm. For images of viral injection confirmation in the VTA, 4 × 4 large images were taken with 10× objective (Plan Apo λ 10×, 0.45 numerical aperture) and z-stack. Images of GFP in the BNST were taken with 10× objective (Plan Apo λ 10×, 0.45 numerical aperture) and z-stack. Acquired images were analyzed in Fiji software and shown as maximum intensity projection of z-stack. For cell counting of eYFP-labeled neurons in the BNST, a region of interest (ROI) was created for each image and saved. DAPI was counted using “Analyze Particles” function in ImageJ after adjusting the threshold and eYFP was counted manually using cell counter function in ImageJ. Final figure preparation was performed in CorelDraw.

### Analysis of intrinsic membrane properties

#### Data exclusion criteria

Exclusion criteria of off-target viral injection and a resting membrane potential more positive than −50 mV were established prior to data collection. Data from mice with off-target viral injection were excluded (*n* = 2). This resulted in 22 mice included in the final analysis (*N* = 22). Neurons with resting membrane potential more positive than −50 mV or did not fire at all in excitability protocol were not further recorded from and excluded from all data analysis. The total number of recorded GFP-positive neurons that were used for electrophysiological characterization was 133 (*n* = 133). Three to 10 neurons were recorded per animal (mean = 6.4 neurons/mouse). For the initial hierarchical clustering and heatmap analysis, a total 128 neurons from 22 mice, each with three evaluated electrophysiological parameters were used. For a more precise hierarchical clustering and heatmap analysis, a total 35 neurons from 17 mice, each with six evaluated electrophysiological parameters were used. In both analysis, neurons that lacked at least one of these parameters were excluded upon creating data matrix.

#### Number of spikes during depolarizing current injection

The number of spikes during depolarizing current injection (excitability: 0 pA to +60 pA for 500 ms; MFR: 50 pA to +750 pA for 1,000 ms) was counted by event detection in Clampfit 11. The number of spikes before a depolarization block occurs was measured as the MFR (Tateno et al., 2004). Also, the specific current step that produced the MFR was considered as one parameter.

#### *I*_h_ (pA/pF)

The difference between peak *I*_h_ and steady-state *I*_h_ during voltage steps from −60 mV to −150 mV was quantified in Clampfit 11 (Szücs et al., 2012). The raw value of *I*_h_ was divided by cell capacitance (pF) to take into account the cell size and to provide current density (pA/pF).

#### Voltage sag (mV)

The voltage sag was measured as the difference between peak voltage sag and steady-state voltage during current steps from −80 pA to −10 pA and was quantified in Clampfit 11.

#### Inter-spike interval (ISI; ms)

For raw inter-spike interval (ISI), “Time of Peak (ms)” and “Interevent interval (ms)” were obtained from Statistics data from excitability protocol (depolarizing current injection from 0 pA to +60 pA in Clampfit 11). For histogram, the bin of ISI was set as 5 ms because minimum ISI was 11.7 ms. The number of ISI that fell within a specific bin was counted. The counts were divided by the sample size of each neuron type for normalization. The first ISI was the earliest recorded ISI at each specific current injection step.

#### First spike latency (FSL; ms)

First spike latency (FSL) was defined as time difference between the peak of the first spike and the depolarizing current injection timing. This raw data was obtained from excitability recording of depolarizing current injection from 0 pA to +60 pA. Since all of the Type “Others” neurons did not fire until 30 pA, this type was excluded from statistical analysis.

#### Rebound burst

The number of spikes during rebound depolarization upon termination of hyperpolarizing current injection (excitability: −80 pA to 0 pA) was counted by event detection in Clampfit 11.

#### Statistics

All analyses were performed with Prism 9 software. Comparisons were achieved by means of analyses of variance without repeated factors (Two-Way-ANOVA, or One-way ANOVA when appropriate). When statistical significance was found in overall ANOVA, *post-hoc* multiple comparisons were made using Tukey test. Data are expressed as the mean ± s.e.m. Statistical significance was set at *P* < 0.05, ****P < 0.0001, ***P < 0.001, **P < 0.01, *P < 0.05; ns, not significant.

### 3D scatter plot

A 3D scatter plot was created in R package rgl with function plot3d utilizing three parameters (“the number of evoked spikes at 60 pA,” “the number of rebound spikes at −80 pA,” and “voltage sag at −80 pA). A total number of 131 neurons from 22 mice were included in the plot.

#### Hierarchical clustering

Two unbiased categorization were generated for comparison of the relationship of the number of parameters used and the quality of categorization. One was with three parameters and the next was with six parameters. In both unbiased analysis, cells that lacked at least one of the parameters were excluded from the data matrix. Both hierarchical clustering were performed using the R (ver.4.1.1) package hclust with option method = ward.D2. A total of 128 neurons from 22 mice were included for the hierarchical clustering and corresponding heatmap analysis with three parameters: “*I*_h_ at −140 mV,” “voltage sag at −80 pA,” “the number of rebound spikes at −80 pA.” The heatmap was generated using the R package ComplexHeatmap. In order to produce more detailed analysis of potential subtypes the number of parameters included within the hierarchical clustering was increased to six parameters: “*I*_h_ at −140 mV,” “voltage sag at −80 pA,” “current step that gives the maximum firing rate,” “first spike latency at 10 pA,” “the number of rebound spikes at −80 pA,” and “first ISI at 40 pA.” These additional parameters help signify the visual difference between the types of neurons. Given that Type “Others” does not have values for “first spike latency at 10 pA” and “first ISI at 40 pA, ” Type “Others” was removed from this analysis. A total of 35 neurons from 17 mice were evaluated for all six parameters and were included for both hierarchical clustering and corresponding heatmap analysis.

## Results

### VTA projecting BNST neurons originate primarily from the caudal BNST

**Figure 1:**
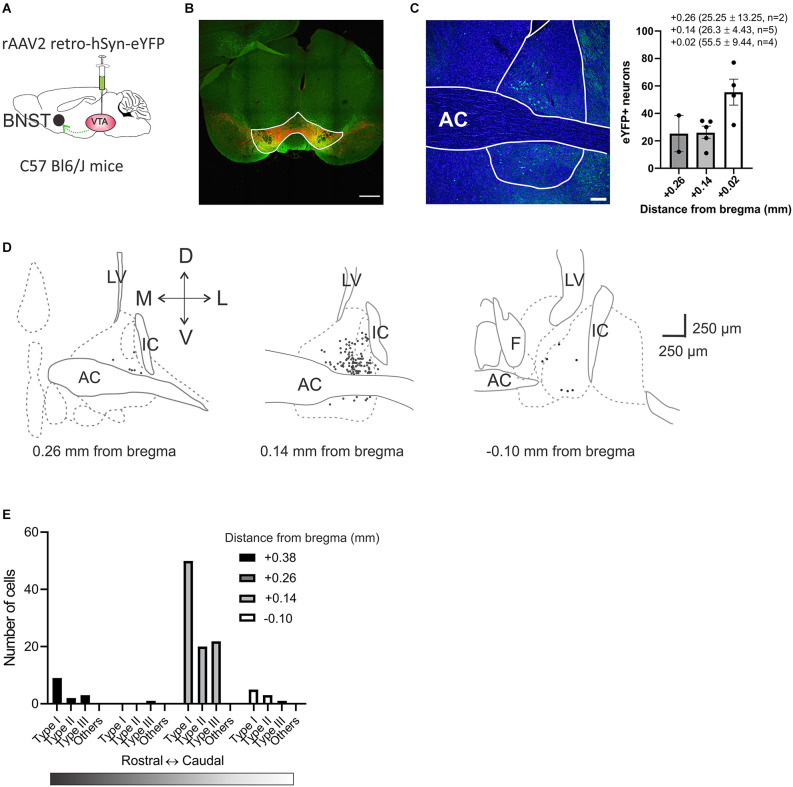
Anatomical distribution of VTA projecting BNST neurons across the BNST. **(A)** Schematic diagram of bilateral viral injection. rAAV2 retro-hSyn-eYFP was injected into the VTA of C57Bl6 male mice. The virus is taken up by axon terminals and transported retrogradely to cell bodies. **(B)** Image shows virally-mediated eYFP expression (green) in VTA injection site with tyrosine hydroxylase (TH, red). Scale bar 500 μm. **(C)** Retrogradely labeled VTA projecting neurons were localized by rAAV2 retro-hSyn-eYFP expression in the BNST. Scale bar 100 μm. **(D)** Anatomical distribution of recording sites of VTA projecting BNST neurons. BNST neurons that project to the VTA neurons were mainly localized in dorsolateral BNST +0.14 mm from bregma (Paxinos and Franklin, 2008). **(E)** Distribution count of recorded neurons across the BNST. AC, anterior commissure; LV, lateral ventricles; IC, internal capsule; F, fornix; BNST, bed nucleus of the stria terminalis; VTA, ventral tegmental area.

Previous recordings of the BNST across species have revealed that the BNST is electrophysiologically diverse (Daniel et al., 2017). To determine the functional diversity within the VTA-projecting BNST neurons in mice, we utilized projection-specific whole-cell patch-clamp recording in coronal slices containing the BNST. To identify the VTA-projecting BNST neurons, a recombinant retrograde adeno-associated virus serotype 2 (rAAV2) carrying enhanced yellow fluorescent protein (eYFP) was injected into VTA (Figure 1A). This allowed for the BNST neurons that project to the VTA to be labeled with eYFP and to be easily visualized during *in vitro* recordings. First, the VTA injection site was confirmed by immunohistochemistry experiments. The VTA was visualized using anti-tyrosine hydroxylase (TH) antibody in red as TH is a marker for dopaminergic neurons (Figure 1B). The injection location was confirmed with co-expression of eYFP carried by retrograde rAAV2 virus and the overlapping TH signal in red in the VTA. We quantified the eYFP-labeling efficiency of the retrograde virus in the BNST. We observed overall 2% of BNST cells counted using DAPI were eYFP-positive meaning that they are VTA-projecting (11 slices from two mice; Figure 1C). The number of eYFP-positive neurons increases toward the caudal region of the BNST; 25.25 ± 13.25 neurons (1.28%) at +0.26 mm from bregma (two slices from two mice), 26.30 ± 4.43 neurons (1.62%) at +0.14 mm from bregma (five slices from two mice), and 55.5 ± 9.44 neurons (2.76%) at +0.02 mm from bregma (four slices from one mouse; Figure 1C). This result is consistent with previous studies suggesting that BNST neurons have a projection to the VTA.

### Functional characterization of VTA-projecting BNST neurons in mice reveals four types of neurons

**Figure 2:**
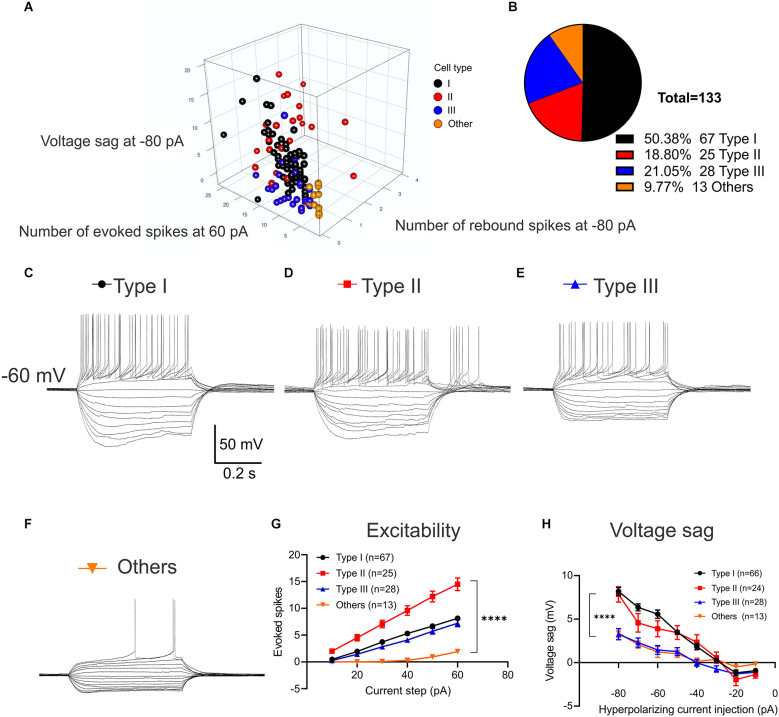
Classification of VTA-projecting BNST neuron activity in response to a series of current injections. Neurons were preliminarily clustered according to firing phenotypes; including excitability, voltage sag and the presence of a rebound spike following hyperpolarization. **(A)** 3D scatter plot of raw values summarizing clusters (*n* = 131 neurons from *N* = 22 mice). **(B)** Pie chart showing the percentage of VTA-projecting BNST neurons categorized by neuronal type (*n* = 133 neurons from *N* = 22 mice). **(C)** Sample traces of voltage response to current steps from −80 pA to +60 pA for 500 ms in Type I neurons. **(D)** Type II neurons with rebound burst at termination of hyperpolarizing current injection. **(E)** Type III neurons without voltage sag. **(F)** Another type of Type “Others” neurons with late firing. Scale bar: 50 mV and 0.2 s. **(G)** Type II neurons exhibit higher number of spikes with the same size current injection compared to Type I, III, and Type “Others” neurons. A two-way ANOVA revealed a significant interaction between the effect of types and current steps on the number of evoked spikes, *F*_(15,774)_ = 6.308, *P* < 0.0001. Tukey *post-hoc* test revealed that Type II neurons had significantly higher number of evoked spikes at 20–60 pA current injection than other three neuron types (adjusted *P* = 0.0006, vs. Type I; adjusted *P* = 0.0004, vs. Type III; adjusted *P* = < 0.0001, vs. Type “Others” at 20 pA; adjusted *P* < 0.0001, vs. Type I; adjusted *P* < 0.0001, vs. Type III; adjusted *P* < 0.0001, vs. Type “Others” at 60 pA). **(H)** Type I and Type II neurons exhibit a significantly larger voltage sag compared to Type III and Type “Others.” A two-way ANOVA revealed that there was a significant interaction between the effect of types and current steps on voltage sag, *F*_(21,1016)_ = 4.634, *P* < 0.0001. Tukey *post-hoc* test revealed that Type I neurons had significantly larger voltage sag (mV) than Type III and Type “Others” at −80 pA through −50 pA steps (adjusted *P* < 0.0001, vs. Type III; adjusted *P* < 0.0001, vs. Type “Others” at −80 pA; adjusted *P* = 0.0039, vs. Type III; adjusted *P* = 0.0263, vs. Type “Others” at −50 pA). Type II neurons has significantly larger voltage sag than Type III at −80 pA through −40 pA step (adjusted *P* < 0.0001 at −80 pA; adjusted *P* = 0.0188 at −40 pA). Type I and Type II neuron were not significantly different throughout hyperpolarizing current injection of −80 pA to −10 pA (adjusted *P* = 0.9633 at −80 pA; adjusted *P* = 0.9203 at −10 pA). Error bars are mean ± SEM. ****P < 0.0001.

Three types of BNST neurons that differ in electrophysiological characteristics are well-studied in rats, mice, and primates. However, the majority of studies in mice do not specify projections or have limited power due to a small sample size (Silberman et al., 2013) which has resulted in inconsistencies in the cell types present. Thus, we conducted a series of whole-cell patch-clamp recordings of multiple parameters of eYFP-positive BNST neurons in a larger sample size to functionally characterize the VTA-projecting BNST neurons in mice. The location of recorded neurons and the number of each type across the rostral-caudal axis are shown (Figures 1D,E). We first evaluated excitability through a series of voltage responses to a 500 ms current injections from −80 pA to +60 pA at 10 pA increments. From this, we measured the number of spikes elicited at each current step, the size of the voltage sag, and the presence of a rebound spike following hyperpolarization. Based on these measurements, we were able to initially classify four types of VTA-projecting BNST neurons (Figures 2A–F). We confirmed three types of neurons: neurons with a large voltage sag and no rebound were categorized as Type I (*n* = 67), neurons with voltage sag and rebound spikes were categorized as Type II (*n* = 25), and neurons with no voltage sag and no rebound spikes were categorized as Type III (*n* = 28) as previously found in rats (Hammack et al., 2007; Figures 2C–E). We also identified a fourth group of neurons (*n* = 13) that have a significantly lower firing rate than Type I, Type II, and Type III neurons (adjusted *P* = 0.0001, vs. Type I; adjusted *P* < 0.0001, vs. Type II; adjusted *P* = 0.02, vs. Type III at +30 pA step; Figure 2E). This finding is consistent with previously reported Type “Others” or late firing (LF) classification which has a late-firing phenotype (Rodríguez-Sierra et al., 2013; Silberman et al., 2013; Daniel et al., 2017).

Upon classification, we found a significant main effect of neuron type on the number of evoked spikes (Two-Way ANOVA, *F*_(3,774)_ = 148.9, *P* < 0.0001) and a significant main effect of current steps (Two-Way ANOVA *F*_(5,774)_ = 88.98, *P* < 0.0001). These main effects were qualified by a significant interaction between the effect of types and current steps, *F*_(15,774)_ = 6.308, *P* < 0.0001 (Figure 2G). *Post-hoc* tests revealed that Type II neurons had a significantly higher number of evoked spikes at 20–60 pA current injection than the other three cell types (Tukey *post-hoc* adjusted *P* = 0.0006, vs. Type I; adjusted *P* = 0.0004, vs. Type III; adjusted *P* = <0.0001, vs. Type “Others” at 20 pA; adjusted *P* < 0.0001, vs. Type I; adjusted *P* < 0.0001, vs. Type III; adjusted *P* < 0.0001, vs. Type “Others” at 60 pA). We found that Type I and Type III neurons were not significantly different at 10–60 pA current injection (adjusted *P* = 0.9842 at 10 pA; adjusted *P* = 0.4683 at 60 pA). Interestingly, Type “Others” only fired at larger depolarizing current injections with a single spike (Figure 2G).

Next, we quantified the voltage sag based on the difference between the peak level of voltage sag at the beginning of the hyperpolarizing current injection and the steady-state level at the end of the hyperpolarizing current injection (Figure 2H). Analysis of effect of neuronal types and current steps on voltage sag revealed that there was a significant main effect of types, (Two-Way ANOVA, *F*_(3,1016)_ = 39.54, *P* < 0.0001) and significant main effect of current steps, (Two-Way ANOVA, *F*_(7,1016)_ = 64.18, *P* < 0.0001). These main effects were qualified by a significant interaction between the effect of types and current steps, (Two-Way ANOVA, *F*_(21,1016)_ = 4.634, *P* < 0.0001). Tukey *post-hoc* test revealed that Type I neurons had significantly larger voltage sag (mV) than Type III and Type “Others” at −80 pA through −50 pA steps (adjusted *P* < 0.0001, vs. Type III; adjusted *P* < 0.0001, vs. Type “Others” at −80 pA; adjusted *P* = 0.0039, vs. Type III; adjusted *P* = 0.0263, vs. Type “Others” at −50 pA). Type II neurons have significantly larger voltage sag than Type III at −80 pA through −40 pA step (adjusted *P* < 0.0001 at −80 pA; adjusted *P* = 0.0188 at −40 pA). Type I and Type II neurons are not significantly different throughout hyperpolarizing current injection of −80 pA to −10 pA (adjusted *P* = 0.9633 at −80 pA; adjusted *P* = 0.9203 at −10 pA).

While Type I and Type III showed similar excitability (adjusted *P* = 0.9842 at 10 pA; adjusted *P* = 0.4683 at 60 pA) which does not aid in classification, Type I has significantly larger voltages sag at −80 pA through −50 pA steps (adjusted *P* < 0.0001, vs. Type III at −80 pA; adjusted *P* = 0.0039, vs. Type III at −50 pA) which was used to separate the two neuronal types.

### VTA projecting BNST neuronal types exhibit different frequency adaptation

**Figure 3:**
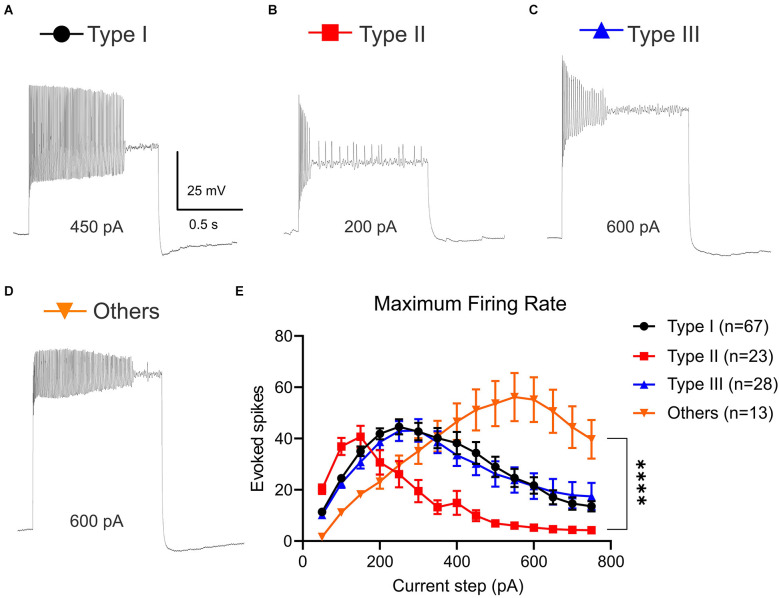
Maximum firing rate (MFR) of VTA projecting BNST neurons. Current was injected from 50 pA to 750 pA for 1,000 ms. **(A–D)** Sample traces of each neuron type at current step that exhibits MFR (Type I, 450 pA; Type II, 200 pA, Type III, 600 pA, and Type “Others”, 600 pA). **(E)** Type II neurons reached depolarization block faster than Type I, Type III, and Type “Others”. A two-way ANOVA revealed that there was a significant interaction between the effect of types and current steps, *F*_(42,1905)_ = 5.359, *P* < 0.0001. Tukey *post-hoc* test revealed that at +500 pA and +550 pA steps, Type “Others” had significantly higher MFR than Type I, Type II, and Type III (adjusted *P* = 0.0021, vs. Type I; adjusted *P* < 0.0001, vs. Type II; adjusted *P* = 0.0020, vs. Type III). ****P < 0.0001.

The temporal responsiveness of neurons depends on the ability to reliably generate and propagate action potentials. One way to evaluate the rate of action potential generation is to measure MFR. Because Type I-III neurons show different frequency adaptation (Figures 2C–E), we measured MFR to calculate when each neuronal type reached depolarization block or firing rate plateau (Tateno et al., 2004; Figures 3A–D). Using depolarizing current steps from +50 pA to +750 pA, we found distinct profiles of MFR among Type I, Type II, Type III, and Type “Others” (Figures 3A–D). There was a significant main effect of neuronal types on number of evoked spikes, (Two-Way ANOVA, *F*_(3,1905)_ = 40.82, *P* < 0.0001) and significant main effect of current steps, (Two-Way ANOVA, *F*_(14,1905)_ = 9.041, *P* < 0.0001; Figure 3E). These main effects were qualified by a significant interaction between the effect of types and current steps (Two-Way ANOVA, *F*_(42,1905)_ = 5.359, *P* < 0.0001). While *post-hoc* test revealed Type I and Type III neurons were not significantly different throughout current steps from +50 pA to +750 pA (adjusted *P* = 0.9959 at +50 pA; adjusted *P* = 0.3329 at +750 pA), we saw a significant difference in Type II and other neuronal types. We found that Type II neurons reached depolarization block faster than Type I, Type III, and Type “Others” (Figures 3A–D). This is demonstrated by Type II neurons increasing quickly with a significantly higher MFR at +100 pA and +150 pA compared to Type “Others” (adjusted *P* = 0.0063 at +100 pA; adjusted *P* = 0.0237 at +150 pA), but rapidly decreasing and becoming significantly lower than Type I and Type III at +250 pA and +300 pA steps (adjusted *P* = 0.0047, vs. Type I; adjusted At +350 pA and +400 pA). Type II neurons had significantly lower MFR than Type I, Type III, and Type “Others” at +350 pA and +400 pA step (at +350 pA: adjusted *P* < 0.0001, vs. Type I; adjusted *P* = 0.0005, vs. Type III; adjusted *P* = 0.0027, vs. Type “Others”; at +400 pA adjusted *P* = 0.0001, vs. Type I; adjusted *P* = 0.0196, vs. Type III, adjusted *P* = 0.0004, vs. Type “Others”).

Interestingly, Type “Others” can fire at larger depolarizing current injections and exhibit a higher MFR. At +500 pA and +550 pA steps, Type “Others” had significantly higher MFR than Type I, Type II, and Type III (adjusted *P* = 0.0021, vs. Type I; adjusted *P* < 0.0001, vs. Type II; adjusted *P* = 0.0020, vs. Type III). At +600 pA through +750 pA, Type “Others” had a significantly higher MFR than Type I, Type II, and Type III (+600 pA: adjusted *P* < 0.0001, vs. Type I; adjusted *P* < 0.0001, vs. Type II; adjusted *P* < 0.0001, vs. Type III; adjusted *P* < 0.0001, vs. Type I; adjusted *P* < 0.0001, vs. Type II; +650 pA adjusted *P* = 0.0003, vs. Type III, at +700 pA adjusted *P* < 0.0001, vs. Type I; adjusted *P* < 0.0001, vs. Type II; adjusted *P* = 0.0030, vs. Type III at; +750 pA adjusted *P* = 0.0009, vs. Type I; adjusted *P* < 0.0001, vs. Type II; adjusted *P* = 0.0192, vs. Type III).

The varied MFR indicates that the neuron-types are potentially differentially tuned for either fast signal transmission, whereas other signals play a more integrative role.

### Dissimilar hyperpolarization activated current (*I*_h_) in VTA projecting BNST neuronal types

**Figure 4:**
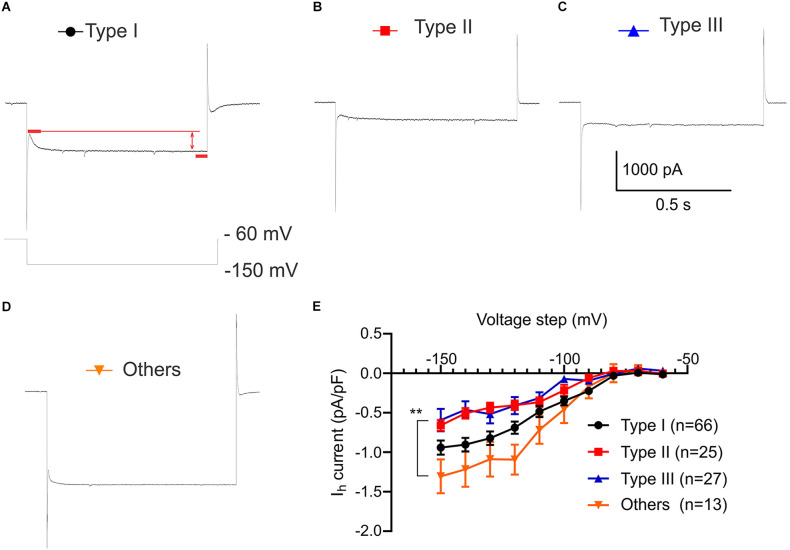
Hyperpolarization activated current (*I*_h_) (pA/pF) of VTA projecting BNST neurons. *I*_h_ recording protocol of voltage step from −150 mV to −60 mV. **(A–D)** Sample trace of *I*_h_ of Type I-III and Type “Others” at −150 mV step. **(E)** Comparison of *I*_h_ shows that Type “Others” has a significantly larger *I*_h_ compared to Type II and Type III*. I*_h_(pA) was normalized by capacitance (pF). A two-way ANOVA revealed that there was a significant interaction between the effect of neuronal types and voltage steps, *F*_(27,1270)_ = 1.915, *P* = 0.0033. Tukey *post-hoc* test revealed that Type “Others” had significantly larger *I*_h_ density (pA/pF) than Type II and Type III at −120 mV through −150 mV voltage steps (adjusted *P* = 0.0001, vs. Type II; adjusted *P* < 0.0001, vs. Type III at −120 mV; adjusted *P* = 0.0003, vs. Type II; adjusted *P* < 0.0001, vs. Type III at −150 mV). **P < 0.001.

Hyperpolarization-activated current (*I*_h_) is an inward current generated by the hyperpolarization-activated cyclic nucleotide gated cation channels which function to regulate neuronal excitability, synaptic activities, and resting membrane potential (Kase and Imoto, 2012). Since voltage sag is considered to be driven by hyperpolarization activated currents (Robinson and Siegelbaum, 2003), we measured *I*_h_ in voltage-clamp configuration using hyperpolarized voltage step from −150 mV to −60 mV (Figures 4A–D). A two-way ANOVA was performed to analyze the effect of types and current steps on *I*_h_ density (pA/pF) during hyperpolarization voltage steps. There was a significant main effect of neuronal types, *F*_(3,1270)_ = 28.07, *P* < 0.0001 and significant main effect of voltage steps, *F*_(9,1270)_ = 53.93, *P* < 0.0001. This main effect was qualified by a significant interaction between the effect of types and voltage steps, *F*_(27,1270)_ = 1.915, *P* = 0.0033 (Figure 4E). Consistent with the largest voltage sag observed in Type I neurons, Type I has a significantly larger *I*_h_ density than Type II and Type III at −120 mV through −150 mV voltage steps (Figure 4E; At −120 mV adjusted *P* = 0.0483, vs. Type II; adjusted *P* = 0.0446, vs. Type III; at −150 mV adjusted *P* = 0.0492, vs. Type II; adjusted *P* = 0.0063, vs. Type III). While Type II express a voltage sag and Type III do not, neither show a significantly different *I*_h_ at −60 mV through −150 mV voltage step (adjusted *P* = 0.9925 at −60 mV; adjusted *P* = 0.9587 at −150 mV). In contrast, *post hoc* analysis revealed that Type “Others” had significantly larger *I*_h_ density (pA/pF) compared to Type II and Type III at −120 mV through −150 mV voltage steps despite having no voltage sag, indicating different ionic mechanisms (Figure 4E; adjusted *P* = 0.0001, vs. Type II; adjusted *P* < 0.0001, vs. Type III at −120 mV; adjusted *P* = 0.0003, vs. Type II; adjusted *P* < 0.0001, vs. Type III at −150 mV).

### VTA projecting BNST neuronal types exhibit distinct spike patterns

**Figure 5:**
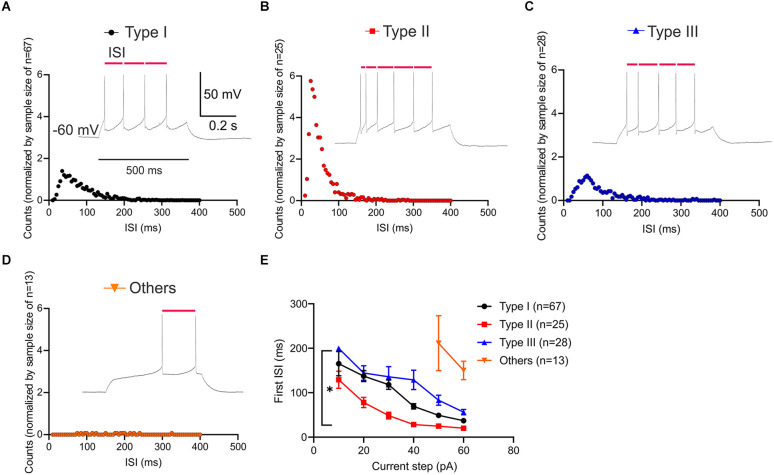
Interspike interval (ISI; ms) patterns of VTA projecting BNST neurons. **(A–D)** Histogram of ISI and sample traces of each neuronal type at 60 pA current step for 500 ms (inset). Scale bar: 50 mV, 0.2 s. The horizontal axis is for the time between each spike and the vertical axis is for the number of intervals at an ISI value normalized by sample size. **(E)** First ISI comparison among groups. Type II neurons have significantly higher number of shorter ISI, as compared to Type I and Type III (adjusted *P* = 0.0003, vs. Type I; adjusted *P* = 0.0033, vs. Type III at 20 pA). *P < 0.05.

The temporal profiles of neuronal firing activity are known to differentially encode neural information. To characterize the spike pattern of each neuronal type, inter-spike interval (ISI) was measured (Figures 5A–D). Type II neurons exhibit a higher number of short ISIs (25 ms), reflecting the nature of low-threshold bursts (Figure 5B). Analysis of the first ISI was performed to compare Type I, Type II, and Type III (Figure 5E). Type “Others” was excluded due to a lack of firing at lower current steps (+10 pA to +40 pA). This analysis revealed that there was a significant main effect of neuronal type (Two-way ANOVA: *F*_(2,520)_ = 29.43, *P* < 0.0001) and significant main effect of current steps, *F*_(5,520)_ = 27.49, *P* < 0.0001. This main effect was qualified by a significant interaction between the effect of types and current steps, *F*_(10,520)_ = 2.026, *P* = 0.0290. Type II neurons have significantly shorter first ISI compared to Type I and Type III at current injection of +20 pA through +40 pA (adjusted *P* = 0.0003, vs. Type I; adjusted *P* = 0.0033, vs. Type III at 20 pA; adjusted *P* = 0.0048, vs. Type I; adjusted *P* < 0.0001, vs. Type III at 40 pA). Type III neurons had significantly longer first ISI than Type I at +40 pA and +50 pA (adjusted *P* < 0.0001 at +40 pA; adjusted *P* = 0.0148 at +50 pA). These differential temporal patterns likely convey information at different timescales.

**Figure 6:**
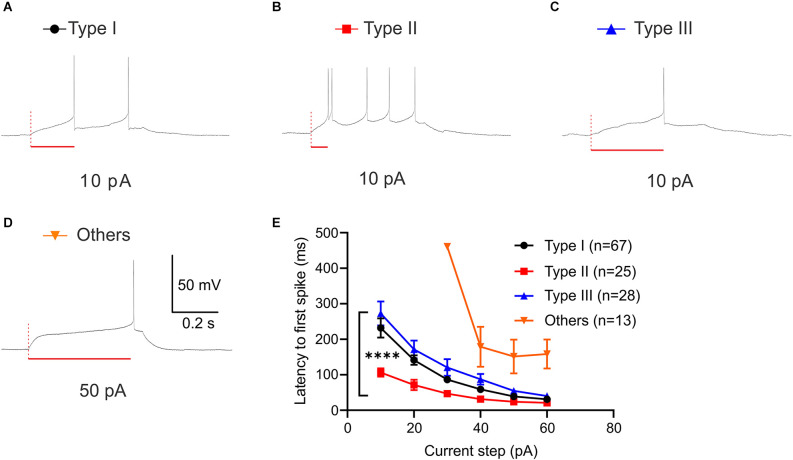
First spike latency of VTA projecting BNST neurons. **(A–D)** Sample traces of first spike latency at 10 pA (Type I-III) and 50 pA (Type “Others”). **(E)** Type II neurons exhibit a shorter spike latency compared to Type I and Type III. A two-way ANOVA revealed that there was a significant interaction between the effect of neuronal types and current steps, *F*_(10,580)_ = 3.967, *P* < 0.0001. Tukey *post-hoc* test revealed that Type II neurons had significantly shorter latency to first spike than Type I and Type III at +10 pA through +30 pA (adjusted *P* < 0.0001, vs. Type I; adjusted *P* < 0.0001, vs. Type III at +10 pA; adjusted *P* < 0.0001, vs. Type I; adjusted *P* < 0.0001, vs. Type III at +20 pA; adjusted *P* = 0.0142, vs. Type I; adjusted *P* < 0.0001, vs. Type III at +30 pA). ****P < 0.0001.

Latency to the first spike is defined as the time delay until the neuron elicits its first action potential. As Type II neurons show low-threshold bursting in response to depolarizing current injection, we quantified the latency to first spike (FSL) across neuronal types. Using current step protocol (−80 pA to +60 pA), we found that each neuronal type demonstrates a different FSL (Figures 6A–D). Since all of the Type “Others” neurons did not fire until 30 pA, this type was excluded in statistical analysis. A two-way ANOVA revealed that there was a significant main effect of neuronal types, *F*_(2,580)_ = 47.72, *P* < 0.0001 and significant main effect of current steps on latency to first spike (ms), *F*_(5,580)_ = 60.42, *P* < 0.0001 (Figure 6E). This main effect was qualified by a significant interaction between the effect of types and current steps, *F*_(10,580)_ = 3.967, *P* < 0.0001. Tukey *post-hoc* test revealed that Type I and Type III neurons were not significantly different throughout the current steps +10 pA to +60 pA except for +30 pA (adjusted *P* = 0.0486 at +30 pA). Type II neurons had significantly shorter latency to first spike than Type I and Type III at +10 pA through +30 pA (adjusted *P* < 0.0001, vs. Type I; adjusted *P* < 0.0001, vs. Type III at +10 pA; adjusted *P* < 0.0001, vs. Type I; adjusted *P* < 0.0001, vs. Type III at +20 pA; adjusted *P* = 0.0142, vs. Type I; adjusted *P* < 0.0001, vs. Type III at +30 pA). Type II neurons had significantly shorter latency to first spike than Type III, but not Type I at +40 pA (adjusted *P* = 0.1232, vs. Type I; adjusted *P* = 0.0020, vs. Type III). Together, these differential responsive properties demonstrate that there are four distinct functional types of BNST neurons that project to the VTA in mice.

### Membrane properties are different across VTA projecting BNST neuronal types

**Figure 7:**
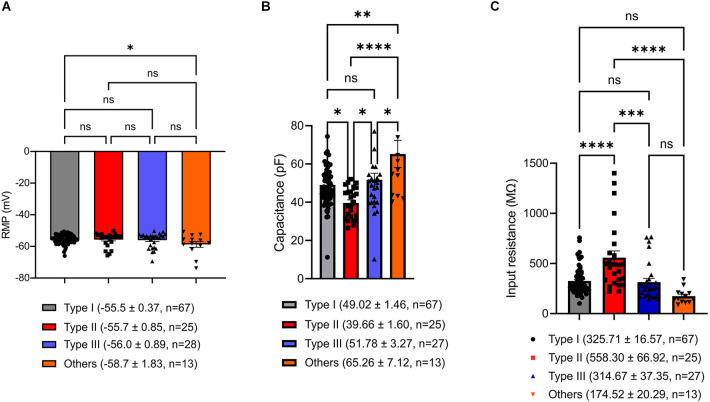
Passive membrane properties of VTA projecting BNST neurons. **(A)** Resting membrane potential (RMP). Type “Others” exhibit significantly hyperpolarized RMP compared to Type I (adjusted *P* = 0.0482). **(B)** Capacitance. Type “Others” have significantly larger capacitance compared to Type I, Type II, and Type III (adjusted *P* = 0.0018, vs. Type I; adjusted *P* < 0.0001, vs. Type II; adjusted *P* = 0.0333, vs. Type III). **(C)** Input resistance. Type II neurons have significantly higher input resistance than Type I, Type III, and Type “Others” (adjusted *P* < 0.0001, vs. Type I; adjusted *P* = 0.0001, vs. Type III; adjusted *P* < 0.0001, vs. Type “Others”). *P < 0.05, **P < 0.01, ***P < 0.001, ****P < 0.0001, ns, not significant.

**Figure 8:**
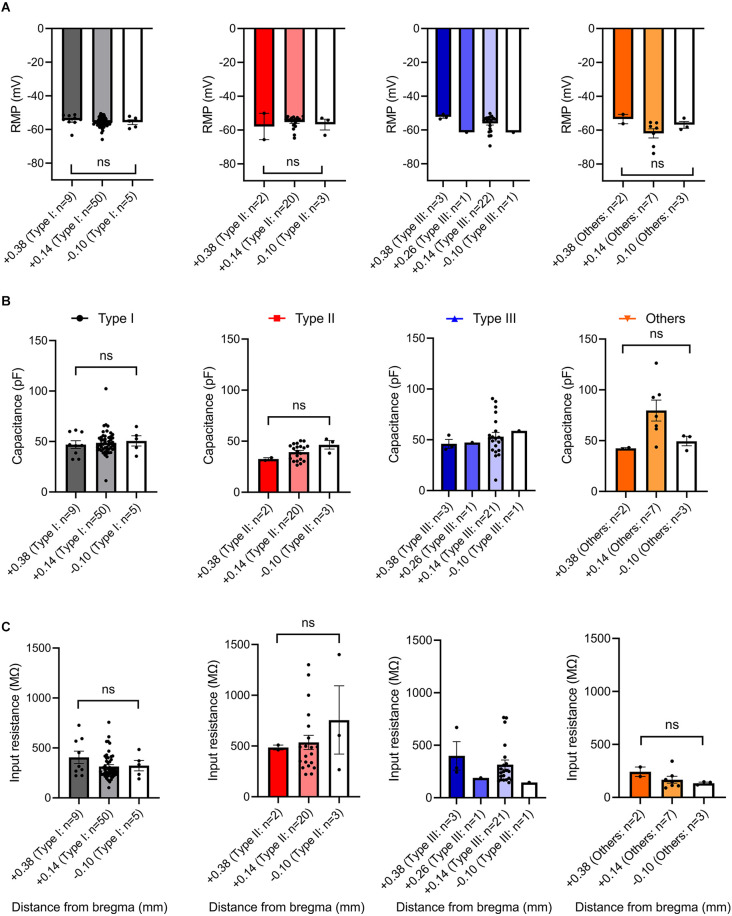
Passive membrane properties of VTA projecting BNST neurons across rostral-caudal axis. **(A)** Resting membrane potential across rostral-caudal axis. **(B)** Capacitance across rostral-caudal axis. **(C)** Input resistance across rostral-caudal axis. ns, not significant.

To seek factors driving clear differences in responses to current injection across neuronal Type I, Type II, Type III, and Type “Others”, we examined passive membrane properties including resting membrane potential (RMP), capacitance, and input resistance. Since the BNST has heterogeneous structure across the rostral-caudal axis (Lebow and Chen, 2016), we also compared these parameters across the rostral-caudal axis of the BNST. A one-way ANOVA revealed that there was no overall significant effect of neuronal types on RMP, *F*_(3,129)_ = 2.328, *P* = 0.0776 (Figure 7A). However, Tukey *post-hoc* test revealed that Type “Others” had significantly more negative RMP than Type I (adjusted *P* = 0.0482). Further, we found no difference in RMP across spatial distribution from rostral to caudal. A one-way ANOVA revealed that there was no significant effect of AP axis on RMP of Type I neurons, *F*_(2,61)_ = 1.719, *P* = 0.1877; Type II neurons, *F*_(2,22)_ = 0.5995, *P* = 0.5578; Type “Others”, *F*_(2,9)_ = 1.288, *P* = 0.3221 (Figure 8A). Type III could not be analyzed since there was only one neuron at +0.26 mm and −0.10 mm from bregma.

Capacitance (pF), an indirect measurement of cell-size, was found to show an overall significant difference among the neuronal types (Figure 7B). A one-way ANOVA revealed that there was an overall significant effect of neuronal types on capacitance (*F*_(3,128)_ = 9.240, *P* < 0.000) with *post-hoc* test revealing that Type “Others” had significantly larger capacitance than Type I, Type II, and Type III (adjusted *P* = 0.0018, vs. Type I; adjusted *P* < 0.0001, vs. Type II; adjusted *P* = 0.0333, vs. Type III). Type II neurons had significantly smaller capacitance than Type I and Type III (adjusted *P* = 0.0331. vs. Type I; adjusted *P* = 0.0160, vs. Type III). Type I and Type III were not significantly different (adjusted *P* = 0.8362). A spatial analysis across AP axis revealed that there was no significant effect of AP axis on capacitance of Type I neurons, *F*_(2,61)_ = 0.1543, *P* = 0.8573; Type II cells, *F*_(2,22)_ = 2.091, *P* = 0.1474; Type “Others”, *F*_(2,9)_ = 3.142, *P* = 0.0923. Type III could not be analyzed since there was only one cell +0.26 mm and −0.10 mm from bregma (Figure 8B).

We also found significant differences in input resistance (MΩ), which reflects the extent to which membrane channels are open. A one-way ANOVA revealed that there was an overall significant effect of neuron type on input resistance (MΩ), *F*_(3,128)_ = 13.44, *p* < 0.0001 (Figure 7C). We found that Type II neurons express a significantly higher input resistance compared to Type I, Type III, and Type “Others” (adjusted *P* < 0.0001, vs. Type I; adjusted *P* = 0.0001, vs. Type III; adjusted *P* < 0.0001, vs. Type “Others”). This is consistent with Type II neurons having the highest excitability and rapid firing. A one-way ANOVA revealed that there was no significant effect of AP axis on input resistance of Type I neurons, *F*_(2,61)_ = 1.719, *P* = 0.1877; Type II neurons, *F*_(2,22)_ = 0.5995, *P* = 0.5578; and Type “Others”, *F*_(2,9)_ = 1.288, *P* = 0.3221. Type III cannot be analyzed since there was only one neuron at +0.26 mm and −0.10 mm from bregma (Figure 8C). These passive properties further inform on the different neuronal types and their functionally distinct roles.

### Unbiased analysis and hierarchical clustering reveals distinct neuronal types

**Figure 9:**
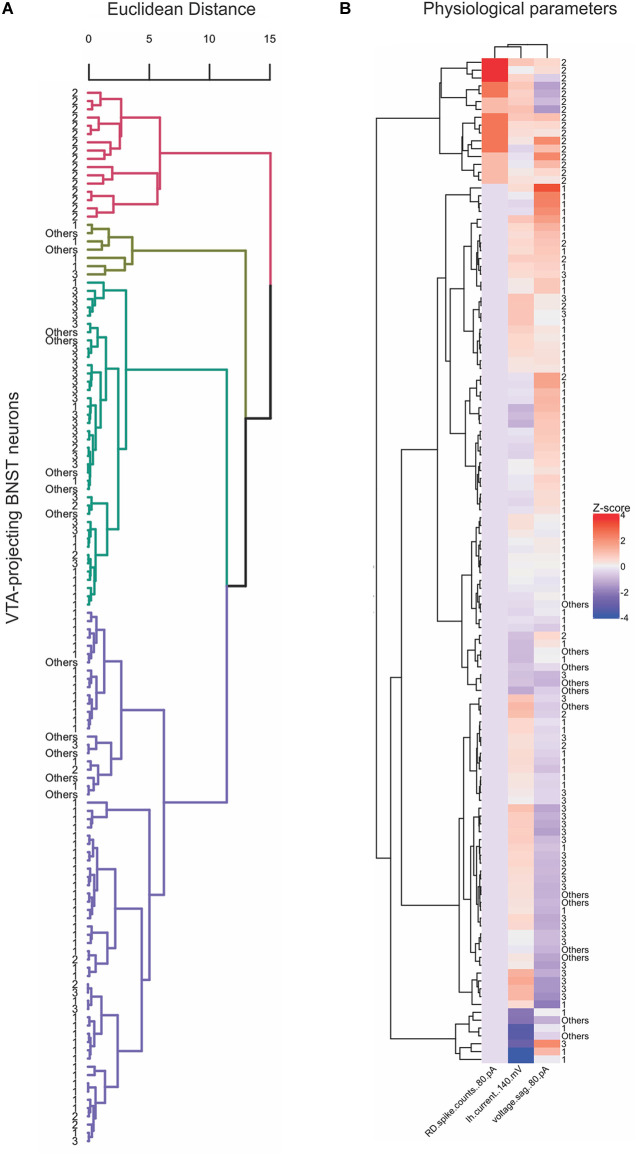
Four preliminary clusters emerge from the unsupervised hierarchical analysis of three electrophysiological parameters of VTA-projecting BNST neurons. **(A)** Dendrogram showing hierarchical clustering using Euclidean distance and Ward’s method (*n* = 128 neurons, *n* = 22 mice). **(B)** Heatmap of neuron features clustered neurons and physiological parameters that were used for clustering.

**Figure 10:**
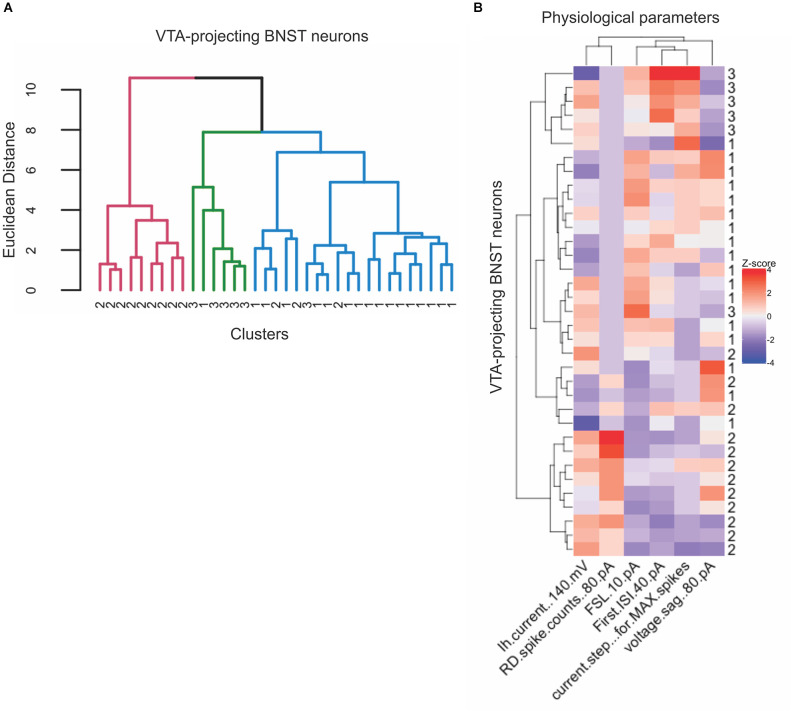
Three neuronal types emerge from hierarchical analysis with six electrophysiological parameters of VTA-projecting BNST neurons. **(A)** Dendrogram showing hierarchical clustering using Euclidean distance and Ward’s method (*n* = 35 neurons = 17 mice). **(B)** Heatmap of neuron features clustered neurons and physiological parameters that were used for clustering.

Previous electrophysiological categorization of the BNST neurons was performed using visual analysis of voltage responses to hyperpolarizing and depolarizing current injection properties (Hammack et al., 2007; Hazra et al., 2011; Yamauchi et al., 2018). To improve categorization, we utilized an unbiased approach to categorize the types of the VTA-projecting BNST neurons in mice through using hierarchical clustering and a heatmap. An advantage of hierarchical clustering is that it does not require a pre-determined number of clusters, therefore providing an unbiased categorization. This analysis can help visualize the relationships between clusters, revealing multiple levels of functional specialization between neuronal types. We first based our analysis on three parameters: *I*_h_ at −140 mV, voltage sag at −80 pA, and number of rebound spikes at −80 pA. Similar to the scatter plot in Figure 2A, the hierarchical clustering of these three electrophysiological properties produced four clusters, with a number of visually identified neurons emerging in different clusters (Figures 9A,B). To produce a more detailed analysis of potential subtypes, we performed our unbiased analysis including six parameters: *I*_h_ at −140 mV, voltage sag at −80 pA, current step that gives the maximum firing rate, first spike latency at 10 pA, number of rebound spikes at −80 pA, and first ISI at 40 pA. These functional parameters were chosen as they encompass more of the unique electrophysiological properties of the VTA-projecting BNST neurons. The recorded neurons were again classified using hierarchical clustering (Figure 10A). Data from neurons identified as Type “Others” were not included in this analysis since they do not have values for all six parameters. Using the six parameters, three clusters emerged which is consistent with our visual categorization: a cluster containing solely Type II (cluster in pink), another cluster containing mainly Type III (cluster in green), and another cluster containing mainly Type I (cluster in blue). Interestingly, within the Type I cluster in blue, we found a small number of Type II (*n* = 3) and Type III neurons (*n* = 1). To further visualize the clustering, specifically the similarities and differences across the categories, we also created a heatmap using the same parameter set (Figure 10B). This visualization allows us to see which parameters are the defining characteristics separating the clusters. In addition, we see that Type II neurons may be further separated into functional clusters based on the presence of voltage sag, while Type I and Type III exhibit multiple shared properties. Together, our unbiased classification based on six parameters from whole-cell patch-clamp recording found four types of VTA-projecting BNST neurons in mice.

## Discussion

The aim of this research was to investigate the electrophysiological diversity of BNST neurons that project to the VTA. This projection is primarily GABAergic and arises primarily from the oval, rhomboid nucleus, interfascicular and transverse regions (Kudo et al., 2012; Jennings et al., 2013; Lebow and Chen, 2016). They are thought to be projecting to the VTA GABAergic interneurons to disinhibit VTA dopaminergic neurons (Kudo et al., 2012, 2014; Jennings et al., 2013; Soden et al., 2020). In this study, we identified four electrophysiologically distinct types of VTA-projecting BNST neurons in mice. We quantified six parameters that can be used for classification. Finally, we performed an unbiased categorization of the neuronal types to visualize similarities and differences between sub-populations.

### Detection of four electrophysiologically distinct sub-types of VTA-projecting BNST neurons in mice

Our whole-cell patch recordings in mice revealed four electrophysiologically distinct neuronal sub-types in the BNST that project to the VTA: Type I, Type II, Type III, and Type “Others”. Without circuit specificity, this is consistent with a study performed in mice, rats, and primates which identified four subtypes (Daniel et al., 2017). However, our result is in contrast to Silberman et al. (2013) which examined the electrophysiological characteristics of the VTA-projecting BNST neurons in mice, but due to small sample size (*n* = 8 neurons), Type III neurons were not observed.

VTA-projecting BNST neurons are arranged topographically. Within the rostral division of the BNST, we found more VTA-projecting BNST neurons in between the level of anterior commissure (+0.14 mm from bregma) and its caudal region (~−0.10 mm), than its rostral region (~+0.38 mm). The proportion of each type, as shown in Figure 1 is, Type I: 50.38% (*n* = 67), Type II: 18.80% (*n* = 25), Type III: 21.05% (*n* = 28), and Type “Others”: 9.77% (*n* = 13). This proportion is in contrast to previous results in mice which examined the entire BNST population without circuit specificity [Type I: 12% (*n* = 7), Type II: 23% (*n* = 13), Type III: 54% (*n* = 30), and Type “Others”: 11% (*n* = 6; Daniel et al., 2017)]. In addition, the proportions of the sub-types are also different from a study performed in VTA-projecting BNST neurons of a rat: [Type I: 4.2% (*n* = 1), Type II: 16.7% (*n* = 4), Type III: 79.2% (*n* = 19; Yamauchi et al., 2018)]. This difference can be explained by circuit specificity and/or animal type used in the study. Neuron selection on the day of recording may also affect the overall proportions. Nevertheless, our results extend previous findings with a larger sample size (*n* = 133) confirming that four electrophysiologically distinct types of VTA-projecting BNST neurons exist in mice.

### The diverse role of BNST-VTA projection

The BNST has been implicated in many functions including reward processing (Dumont et al., 2005; Eiler et al., 2007; Park et al., 2013), anxiety-like behavior (Waddell et al., 2006; Kim et al., 2013), and modulation of stress response (Choi et al., 2007; Radley and Sawchenko, 2011). Among the diverse functions and projections from the BNST, the projection to the VTA is important for stress-reward interaction such as development of drug addiction, stress-induced reinstatement of drug seeking behavior, and withdrawal-induced anxiety. For example, rats that underwent cocaine conditioning and extinction had increased Fos activity of the VTA-projecting BNST neurons after exposure to forced swim test. Pharmacological inactivation of BNST blunted Fos activity in the VTA and reduced reinstatement (Briand et al., 2010). This data suggests that the BNST-VTA connection is required for stress-induced reinstatement. Furthermore, the fact that BNST GABA ≫ midbrain ≫ Amygdala pathway regulates cocaine-induced anxiety highlights the significance of cell type- and projection-specific investigations in translational studies (Tian et al., 2022). Our analysis of the electrophysiological heterogeneity of the VTA-projecting BNST neurons helps expand the foundation to study their cell-type specific role for designing targeted therapeutics for addiction and anxiety.

### Type II VTA-projecting BNST neurons

We found that Type II neurons represent 18.80% of the total BNST neurons projecting to the VTA in mice. This finding extends a previous report which found only a small number of Type II neurons (*n* = 2; Silberman et al., 2013). This evidence suggests a novel function of Type II neurons since Type II neurons have been previously considered as local interneuron modulating Type III neurons because of observed short axon (Larriva-Sahd, 2006; Hammack et al., 2007). In rats, transcriptome analysis of Type II neurons suggests that further differentiation of Type II neurons may be possible based on different neuropeptides (Hazra et al., 2011).

### Type “Others” VTA-projecting BNST neurons

Although categorization of the BNST neurons into three types based on physiological characteristics is well-established, we observed 13 neurons (9.77%) out of 133 recorded neurons that did not fit into three types. This Type “Others” of BNST neurons has been documented previously (Rodríguez-Sierra et al., 2013; Silberman et al., 2013; Daniel et al., 2017; Ch’ng et al., 2019). This Type “Others” features no voltage sag and no fast-inward rectification at the beginning of hyperpolarizing current injection (Rodríguez-Sierra et al., 2013), single spike per depolarizing current spike, and hyperpolarized RMP (Silberman et al., 2013). Our whole-cell patch-clamp recording data of Type “Others” replicated these characteristics and showed unique physiology such as significantly higher maximum firing rate (Figure 3). These neurons differ in that they discharge action potentials at high rates with little or no spike frequency adaptation or attenuation in spike height.

### Unbiased classification of the VTA-projecting BNST neurons

Given the pre-determined classification cluster numbers, the visual categorization might limit our understanding of the diversity of BNST neurons and miss potential distinct functional roles of each cell type. This bias may contribute to the contradictory results of the neuron types observed in previous studies. We first performed an unsupervised classification using three quantified parameters and found four primary clusters in which Type I, Type II, and Type III show a preliminary separation, with further smaller groupings. To increase the precision of our categorization we performed analysis with six parameters which encompass more information about the functionality of the neuronal types: *I*_h_ at −140 mV, voltage sag at −80 pA, current step that gives the maximum firing rate, first spike latency at 10 pA, the number of rebound spikes at −80 pA, and first ISI at 40 pA. These parameters were chosen because they shape the different voltage responses to current injection and have been used to visually categorize the BNST neurons previously.

A heatmap and hierarchical clustering confirmed that six quantified parameters results in a more detailed categorization of three types of BNST neurons, with fewer cluster branches. We found that the hierarchical clustering produces three primary clusters: a cluster solely containing Type II (cluster in pink), another containing mainly Type III (cluster in green), and another containing mainly Type I. Despite the additional three parameters we still found that the Type I cluster, in addition to containing Type I neurons, also contains of Type III (*n* = 1) and Type II neurons (*n* = 3).

Within both heatmaps, we can visualize which parameters define individual clusters. Given the overlap in categorization between clusters of Type I and Type III, it is possible to view the neuronal types as a functional continuum, with shifting ion channel function, rather than clear rigid separation. Indeed, mRNA of diverse ion channel subunits in anterolateral BNST neurons in rats showed differential expression across three types, rather than all-or-none presence of specific mRNA (Hazra et al., 2011).

### Stress changes neuron membrane physiology

The BNST has been found to mediate the sustained response to fear or anxiety, such as increased activation in threat anticipation. Anatomical heterogeneity of the BNST serves to regulate anxiety in opposing ways, being anxiogenic and anxiolytic, with the relative strength of diverse circuits creating a behavioral balance (Kim et al., 2013). Stress alters this balance partially by altering the functionality of neurons. For example, long-term exposure to CRF signaling changes ion channel trafficking (Borodovitsyna et al., 2018). In the BNST, chronic swim stress alters the proportion of neuronal types (Ch’ng et al., 2019) and chronic shock stress differentially affects excitability of each type (Daniel et al., 2019). Our heatmap of VTA-projecting BNST neurons will allow us to see how the ratio of each neuronal type changes and which specific membrane properties are altered upon stress exposure.

While we have functionally identified four distinct neuronal types, a transcriptome analysis of the individual subtypes would help in our understanding of the rigidity of neuronal classification. This applies particularly when the functional type of BNST neurons might be seen as a continuum on a heatmap. Given a study showing that chronic social defeat stress downregulated the expression level of several ion channels in resilient mice (Gururajan et al., 2022), a heatmap classifying BNST neurons combined with transcriptomics will assist in visualizing how the population shifts.

In summary, we introduced an unsupervised method for functional classification of the VTA-projecting BNST neurons, which allows for visualization of shared and dissimilar physiological parameters. Identifying the precise function of different cell types within individual projections is critical to unravel the diverse role of the BNST. A heatmap can expand our understanding of a possible continuum of neuron functioning of the BNST, integrating different inputs and fine-tuning behavioral outputs by population shifts.

## Data Availability Statement

The raw data supporting the conclusions of this article will be made available by the authors, without undue reservation.

## Ethics Statement

The animal study was reviewed and approved by Institutional Animal Care and Use Committee, Hunter College.

## Author Contributions

YM performed all experiments. MS, AS, and YM analyzed data. YM and AF designed experiments and wrote the manuscript. All authors contributed to the article and approved the submitted version.
